# Supplementary material for: Evidence for mirror self-recognition in beluga whales (Delphinapterus leucas)
Source: PLoS One. 2026 May 20;21(5):e0348287. doi: 10.1371/journal.pone.0348287 (PMC13189309; doi:10.1371/journal.pone.0348287)
Supplement: S1 Table — (PDF) [file pone.0348287.s001.pdf]

S1. Ethogram of orientations and behaviors exhibited at mirror or control

Stationing Orientations at Mirror/Control	Description
**Head perpendicular (HPO)**	Head oriented perpendicularly and body is positioned horizontally without any other behavior exhibited
**Horizonal ventral (HVO)**	Horizontal position with ventral side oriented to window without any other behavior exhibited
**Inverted**	Head down and fluke up with ventral side parallel to window in a vertical plane or head perpendicular to window with ventral side up without any other behavior exhibited
**Lateral left eye**	Left eye parallel to the window in a lateral plane without any other behavior exhibited
**Lateral right eye**	Right eye parallel to the window in a lateral plane without any other behavior exhibited
**Vertical left Eye**	Left eye parallel to the window in a vertical plane without any other behavior exhibited
**Vertical right eye**	Right eye parallel to the window in a vertical plane without any other behavior exhibited
**Vertical ventral**	Ventral side parallel to the window in a vertical plane with head facing up without any other behavior exhibited
**Behaviors at Mirror/Control**	
**Barrel roll**	Single or repeated 360º body rotations in horizontal position
**Bubble bite**	Reputedly biting bubbles produced from blowhole or mouth
**Bubble production**	Bubble streams, bursts or scants produced from blowhole or mouth
**Downward head bob (DHB)**	Rapid downward head movement often followed with the rapid opening and closing of mouth
**Firm melon press**	Melon pressed firmly against the window
**Head waggle**	Repeated rapid vertical jiggling of head
**Horizontal head shake**	Shaking the head from side to side
**Jaw clap**	Opening and closing mouth often with by a loud popping noise
**Melon touch**	Light contact of melon against window
**Neck stretch**	Upward stretch of the neck
**Open mouth**	Mouth held wide open for at least 1 sec
**Pec shimmy**	Repeated movement of one or both pectoral fins in vertical orientation
**Regurgitation**	Regurgitation of food
**Semicircular head movement**	Singular or repeated upward and sideward movement of head in arc from left to right or right to left
**Slow downward head dip (SHD)**	Approach from top of the mirror and slowly dipping head down
**Toy manipulation**	Presentation and/or interaction with toy
**Up-down swim**	Repeated continuous movement from top to bottom of mirror
**Upward head jerk**	Quick jerky upward head movement
**Vertical head nod**	Slow repetitive downward head nods
