# Supplementary material for: Evidence for mirror self-recognition in beluga whales (Delphinapterus leucas)
Source: PLoS One. 2026 May 20;21(5):e0348287. doi: 10.1371/journal.pone.0348287 (PMC13189309; doi:10.1371/journal.pone.0348287)
Supplement: S2 Table — (PDF) [file pone.0348287.s002.pdf]

S2. Mark and sham-mark location and order of testing

Individual Tested	Condition	Mark Type	Mark Location on Body
Natasha	Mirror	Early Sham	Above right pec
Natasha	Mirror	Early Sham	Under chin
Natasha	Mirror	Early Sham	Left side of head
Natasha	Control	Early Sham	Below left pec
Natasha	Mirror	Early Sham	Left side of head
Natasha	Control	Early Sham	Under chin
Natasha	Control	Early Sham	Dorsal side of fluke
Natasha	Control	Early Sham	Right side of head
Natasha	Mirror	Early Sham	Across chest
Natasha	Control	Early Sham	Under right pec
Natasha	Control	Early Sham	Behind left ear
Natasha	Mirror	Early Sham	Behind right ear
Natasha	Mirror	Early Sham	Dorsal side of fluke
Natasha	Control	Early Sham	Tongue
Natasha	Control	Early Sham	Across chest
Natasha	Mirror	Early Sham	Tongue
Natasha	Control	Early Sham	Behind left ear
Natasha	Mirror	Early Sham	Right side of melon
Natasha	Mirror	Early Sham	Under chin
Natasha	Mirror	Early Sham	Dorsal side of fluke
Natasha	Mirror	Early Sham	Right side of head
Maris	Control	Early Sham	Right side of head
Natasha	Mirror	Early Sham	Above right pec
Natasha	Mirror	Early Sham	Under Chin
Natasha	Mirror	Early Sham	Behind left ear
Natasha	Mirror	Mark	Right side of melon
Maris	Control	Early Sham	Center of melon
Maris	Control	Early Sham	Right side of head
Natasha	Control	Late Sham	Under chin
Natasha	Mirror	Mark	Left side of melon
Maris	Mirror	Early Sham	Under the chin
Maris	Mirror	Early Sham	Right side of head
Maris	Mirror	Early Sham	Behind left ear
Maris	Mirror	Early Sham	Right pec
Maris	Mirror	Early Sham	Behind right ear
Maris	Mirror	Early Sham	Left shoulder
Maris	Mirror	Mark	Behind Left ear
Maris	Mirror	Mark	Above left pec
Maris	Mirror	Mark	Across chest
Maris	Mirror	Mark	Above right pec

Natasha	Mirror	Late Sham	Behind right ear
Natasha	Mirror	Late Sham	Above left pec
Natasha	Mirror	Mark	Behind right ear
Natasha	Mirror	Mark	Above left pec
Natasha	Mirror	Mark	Across chest
Natasha	Mirror	Mark	Behind left ear
Natasha	Mirror	Mark	Right side of head
Maris	Mirror	Mark	Right side of melon
Maris	Mirror	Mark	Behind right ear
Natasha	Mirror	Mark	Right side of head
Maris	Mirror	Mark	Behind left pec
Natasha	Mirror	Mark	Above left pec
Maris	Mirror	Mark	Under chin
Natasha	Mirror	Mark	Under chin
Maris	Mirror	Mark	Right shoulder
Maris	Mirror	Mark	Left shoulder
Natasha	Mirror	Mark	Left side of melon
Natasha	Mirror	Mark	Left side
Natasha	Mirror	Mark	Right pec
Natasha	Mirror	Mark	Across chest
Maris	Mirror	Mark	Right shoulder
Maris	Mirror	Mark	Left side
Maris	Mirror	Mark	Right side
Maris	Mirror	Mark	Left shoulder
Maris	Mirror	Mark	Right side
