# Supplementary material for: Evidence for mirror self-recognition in beluga whales (Delphinapterus leucas)
Source: PLoS One. 2026 May 20;21(5):e0348287. doi: 10.1371/journal.pone.0348287 (PMC13189309; doi:10.1371/journal.pone.0348287)
Supplement: Attachment — Submitted filename: Response to Reviewers .docx [file pone.0348287.s010.docx]

Response to Reviewers: PLOS One: PONE-D-26-01940

Evidence for mirror self-recognition in beluga whales (*Delphinapterus leucas*)

Dear Dr. Munderloh,

We thank you and both Reviewers 1 and 2 for their time and consideration of our manuscript and for the points and thoughtful suggestions that have been made to make the submission stronger. We have revised our manuscript to address the suggestions provided in the review process. Please find our responses in italics below.

**Editorial Comments**
Please include your full ethics statement in the ‘Methods’ section of your manuscript file. In your statement, please include the full name of the IRB or ethics committee who approved or waived your study, as well as whether or not you obtained informed written or verbal consent. If consent was waived for your study, please include this information in your statement as well.

***Authors’ responses to the Editor***

*As requested, we have included the following Ethics Statement in the Methods section of the manuscript:*

*P 6. lines 126-128: The research protocol was reviewed and verbally approved by the Animal Care Committee of the New York Aquarium of the Wildlife Conservation Society.*

*In addition, we have added a few new references in response to the reviewers’ suggestions and comments and deleted a few. All changes to in-text citations and references have been renumbered as needed. All changes have been highlighted as requested.*

*References added:*

*In our revised manuscript we have added additional references about beluga whale and cetacean vision in response to the comments of Reviewer #2 concerning the ecological and behavioral importance of vision in cetaceans and in belugas specifically. They are numbered as follows:*

*26. Herman LM, Peacock MF, Yunker MP, Madsen CJ. Bottle-Nosed Dolphin: Double-slit pupil yields equivalent aerial and underwater diurnal acuity. Science. 1975;189(4203):650–52. DOI:10.1126/science.1162351*

*27. Racicot RA, Darroch SA, Kohno N. Neuroanatomy and inner ear labyrinths of the narwhal, Monodon monoceros, and beluga, Delphinapterus leucas (Cetacea: Monodontidae). J Anat. 2018;233(4):421-39.* [*https://doi.org/10.1111/joa.12862*](https://doi.org/10.1111/joa.12862)

*28. Mobley JR, Helweg DA. Visual Ecology and Cognition in Cetaceans. In: Thomas JA, Kastlelein RA, editors. Sensory Abilities of Cetaceans: Laboratory and Field Evidence. Plenum Press; 1990. pp. 519–36.* [*https://doi.org/10.1007/978-1-4899-0858-2_36*](https://doi.org/10.1007/978-1-4899-0858-2_36)

*29. O'Corry-Crowe GM. Beluga whale: Delphinapterus leucas. In: Perrin WF, Würsig B, Thewissen JGM, editors. Encyclopedia of marine mammals. San Diego: Academic Press; 2009. pp. 93-6.*

*30. Richard JT, Pellegrini I, Levine R. Belugas (Delphinapterus leucas) create facial displays during social interactions by changing the shape of their melons. Anim Cogn. 2024;27(1):7-15.* [*https://doi.org/10.1007/s10071-024-01843-z*](https://doi.org/10.1007/s10071-024-01843-z)

*References deleted: The following three references in the original submission have been omitted from the revised manuscript due to their redundancy to other papers we cited and the fact they were not peer reviewed although they were cited in other papers. The deleted references were originally numbered as follows:*

32. *Murayama T, Tobayama T. Preliminary study of mental rotation in beluga. In Abstract of the XXIV International Ethological Conference 1995 (Vol. 114).*
33. *Murayama, T. (2008b). Ninchi [Cognition]. In T. Murayama (Ed.), Geiruigaku [Cetology]. Kanagawa: Tokai University Press, pp. 291–326. (Translation from original text in Japanese)*

*45. D’Amore D, Skolnick AJ. Social Awareness as an indicator of self-awareness: the Mead-Cooley model and research methodology in nonhuman primates. J Stud Scholar. 2008;10.*

*We also made a few minor formatting changes such as the addition of a missing period or in one case we added a missing word to the text. We have highlighted these formatting changes in the highlighted version of the text. Below are the pages/lines of these minor changes.*

*P.14 line 339: The word ‘jerk’ was missing from the term ‘upward head jerk’ and we corrected it.*

*P. 15 Table 1: A period was added to the Table title after Table 1. and at the end of the sentence. A superscript ^a^ was added to indicate the footnote.*

*P. 20 line 461: The heading Natasha Mark Test was changed to Mark Test: Natasha*

*P. 20 line 513: The heading Maris Mark test was changed to Mark Test: Maris*

*P. 21 line 496: The word ‘min’ was changes to ‘minutes’ for consistency.*

*P. 21 lines 500-501: The format was corrected for consistency*

***Authors’ Responses to Reviewers***

**Reviewer #1:** I consider this paper to be a valuable contribution to MSR research in a cetacean species for which experimental opportunities are limited. I was pleasantly surprised to learn that the experimental video material dates back to 2001. Although many of the results are descriptive, the data set is clearly worthy of publication, and I appreciate the authors’ efforts in compiling and analyzing these materials.

***Authors’ response:*** *Thank you for your thoughtful comments and suggestions and we have responded with text in italics to the points you have raised and suggested.*

Among the four individuals, two showed clear interest in the mirror. However, given that long-term captive belugas may have previously encountered reflective surfaces such as tank glass, it is quite possible that they already had some degree of mirror-like experience. In addition, considering individual differences, the absence of interest in some is not unexpected.

***Authors’ response:*** *We agree as acknowledged on P 6. lines 135-137 in our original submission.*

Although only Natasha passed the mark test, and only in a single trial, the self-directed behaviors observed in Natasha and Maris provide strong evidence of MSR, I think. Overall, I find the conclusions of this paper to be reasonable.

In recent MSR research, the focus has increasingly shifted from whether a given species is capable of MSR to how MSR is acquired. For example, a mark test study using 9 fish of the cleaner wrasse demonstrated that MSR was established as early as 20 minutes in the fastest individuals, and on average about 80 min after mirror exposure (Sogawa et al. 2025, Scientific Reports). Prior to the establishment of their MSR, cleaner wrasse exhibited social behaviors (aggression to own mirror image) and contingency testing (C-testing) behaviors. These findings suggest that behaviors previously interpreted as C-testing may occur after establishing MSR, and may therefore reflect examination of the properties of the mirror rather than the own mirror image. Similar interpretations have been proposed in many MSR studies, including those on chimpanzees (Chimps will be within 30 min, Povinelli et al. 1993). From this perspective, it would be interesting to consider how long after initial mirror exposure belugas may be able to establish MSR. If possible, I hope the authors to discuss this point in light of the present findings. In Sogawa et al. (2025), individuals of cleaner wrasse were observed to lifting food items (pieces of shrimp) near the water surface in front of the mirror and dropping them to observed their movement. This behavior occurred after establishment of MSR, and is interpreted as an examination of the mirror’s properties. Distinguishing between behaviors directed at the self-image and those directed at the mirror itself may provide additional insight into the interpretation of mirror-related behaviors.

***Authors’ response:*** *As requested, we have included how long after initial mirror exposure the belugas showed behavioral evidence of MSR****.*** *We agree about the importance of not only reporting whether species and individuals exhibit MSR but how it is acquired. Specifically, in our study, as in the majority of past MSR studies, we documented and analyzed the belugas’ behavior during mirror exposure prior to the mark test. Following the basic approach pioneered by Gallup (1970) individuals exposed to a mirror show a progression of changes from social (if mirror naïve) to contingency testing to self-directed. This progression of behavioral change has been generally interpreted as a reflection of an individual’s change in their perception of what they are seeing in the mirror. In these studies, mark tests are conducted after mirror exposure and after the onset of self-directed behaviors indicating MSR. The differences in procedures used in our current study with those used in Sogawa et al. (2025) in which fish were first exposed to a mirror when marked, preclude a direct comparison in the time of onset of MSR. We have added the following paragraph to the manuscript that states the time from initial mirror exposure to the onset of MSR as evidenced by the onset of self-directed behaviors. We have also expanded our previous description of the first self-directed behaviors exhibited.*

*P. 16 lines 362-370: Both whales exhibited self-directed behaviors during the second mirror session after 2 hours and 11 minutes of prior mirror exposure. The first self-directed behavior by Maris was bubble bite play when alone and close to the mirror. During this event, Natasha moved behind Maris and Maris quickly departed from the mirror. Natasha then moved close to the mirror and engaged in bubble bite play. During this activity the whales produced bubbles from their blowhole or mouth and then bit the bubbles (S1 Video). Bubble bite play was the most frequent self-directed behavior to emerge in the mirror condition by both Natasha and Maris. This behavior was reported as self-directed behavior in two past studies of bottlenose dolphins (Tursiops truncatus) (Reiss & Marino, 2001; Morrison & Reiss, 2018). A wide variety of other self-directed behaviors were observed throughout the mirror exposure sessions.*

*As you mentioned, Sowaga et al. (2025) observed the behavior of the cleaner wrasse lifting and dropping food items near the water surface in front of mirror after MSR and interpreted the behavior as testing the reflective properties of the mirror. During the mirror exposure period prior to MSR, individuals have the opportunity to learn the contingencies of mirror use that would include learning about the reflective properties of the mirror when viewing objects, themselves, and themselves interacting with objects. Individuals can learn there is a one-to-one relationship with what they are doing and what they observe reflected in the mirror. In our original manuscript we have a discussion of our findings in which four different behaviors, originally categorized as contingency-testing, continued to be observed after the belugas began exhibited self-directed behaviors and we referred to these as (CTSD) (P. 25-26 lines 599-611). In MSR studies the demarcation line between contingency testing versus self-directed behaviors may be unclear and therefore, as we stated in our original manuscript, to be conservative, the onset of such repetitive and unusual behaviors were initially categorized as contingency-testing. However, these behaviors may function later as self-directed if they continue to be exhibited once other self-directed behaviors begin and continue to be exhibited and the categorization/interpretation of these behaviors is dependent on the temporal and behavioral context in which it occurs (as reported in Povinelli et al. (1993) and Morrison & Reiss (2018)).*

Finally, I note that the citation style in the reference list was not entirely consistent, please make them in the form.

***Authors’ response:*** *In the reference list we corrected the citations so they are all consistent in form.*

**Reviewer #2:** Consider the role of ecology when it comes to mark saliency in Beluga whales. Beluga ecology does not rely on vision as a primary sensory modality the way primates and birds do. Ecological context may be useful when comparing cetacean species tested, in particular to bottlenose dolphins where there is evidence of capacity. Kohda et al (2012) demonstrated the importance of a species ecological context with cleaner wrasse who are biologically wired to attend to unusual visual marks.

Consider the weakness of using a mark test relying on vision in a species where there may not be an ecologically relevant reason for them to attend to the mark. This is where tests for mark saliency such as in Prior et al. (2008) in magpies become useful to demonstrate the species show interest in the mark. Consider referencing papers that have explored mark tests in alternative modalities to address this possibility (eg the olfactory test developed by Cazzolla Gatti (2016) for dogs).

***Authors’ response:*** *We thank you for your perspectives on the importance of a species’ behavioral ecology and using methods appropriate to each species in tests of MSR. We agree and these are indeed important points regarding the role of ecology and species sensory systems when it comes to the mark test. In this study we used methods that have been successfully used in previous tests with dolphins (Reiss & Marino, 2001; Morrison & Reiss, 2018) to ascertain if they have the capacity for visual self-recognition in a mirror. And –the belugas in this study demonstrated the progression from contingency testing to self-directed behaviors and one of the whales passed the mark test. However, based on your comments, it became clearer to us that we needed to provide more information and cite additional literature about the visual prowess of belugas and other cetacean species and that vision plays an important social and ecological role for this large-brained highly social aquatic mammals.*

*In the introduction we have added the following:*

*P. 4 lines 88-96: “Cetaceans are renowned for their hearing and echolocation ability, but their vision was long assumed to be poor. Research has shown that cetaceans do in fact possess strong visual acuity both above and below water [26], that belugas have good binocular vision [27], and that vision plays an important ecological role among cetaceans in prey capture, predator evasion and social interactions [28]. Vision in belugas may be of particular importance among cetaceans due to their unique ability to manipulate the shape of their melon for facial displays in social interactions [27,29,30]. Belugas demonstrate advanced cognitive abilities involving both their visual and auditory sensory systems across many domains similar to the capacities reported for apes, dolphins and elephants.”*

*In the discussion we have added the following:*

*P. 28-29 lines 681-685*: *"It has been reported that the thicker and more anteriorly oriented frontal groves/optic canals in the beluga eye suggest good binocular vision [27]. It has also been reported that belugas show a left eye over right eye preference when inspecting objects [58] which differs from our findings of a right eye preference when viewing self."*

*In the revised manuscript we have added the following references concerning the ecological and behavioral importance of vision in cetaceans and in belugas specifically. They are numbered as follows:*

*26. Herman LM, Peacock MF, Yunker MP, Madsen CJ. Bottle-Nosed Dolphin: Double-slit pupil yields equivalent aerial and underwater diurnal acuity. Science. 1975;189(4203):650–52. DOI:10.1126/science.1162351*

*27. Racicot RA, Darroch SA, Kohno N. Neuroanatomy and inner ear labyrinths of the narwhal, Monodon monoceros, and beluga, Delphinapterus leucas (Cetacea: Monodontidae). J Anat. 2018;233(4):421-39.* [*https://doi.org/10.1111/joa.12862*](https://doi.org/10.1111/joa.12862)

*28. Mobley JR, Helweg DA. Visual Ecology and Cognition in Cetaceans. In: Thomas JA, Kastlelein RA, editors. Sensory Abilities of Cetaceans: Laboratory and Field Evidence. Plenum Press; 1990. pp. 519–36.* [*https://doi.org/10.1007/978-1-4899-0858-2_36*](https://doi.org/10.1007/978-1-4899-0858-2_36)

*29. O'Corry-Crowe GM. Beluga whale: Delphinapterus leucas. In: Perrin WF, Würsig B, Thewissen JGM, editors. Encyclopedia of marine mammals. San Diego: Academic Press; 2009. pp. 93-6.*

*30. Richard JT, Pellegrini I, Levine R. Belugas (Delphinapterus leucas) create facial displays during social interactions by changing the shape of their melons. Anim Cogn. 2024;27(1):7-15.* [*https://doi.org/10.1007/s10071-024-01843-z*](https://doi.org/10.1007/s10071-024-01843-z)

In the limitations the author’s state, “It would have been preferrable to employ a totally non-reflective control rather than a semi-reflective control.” It may be worth recontextualising the role of reflective surfaces when it comes to captive aquatic animals, as their exposure and experience to reflective surfaces may be a key detail impacting the promising results in captive species. Studies where animals are given the mark test after a set number of hours without the prerequisite emergent self-directed behaviours often fail. Too much exposure to reflective surfaces means that the once novel properties may no longer elicit exploratory or curious behaviours. A lack of social behaviours towards a reflection could indicate understanding of mirror properties, but if they do not display self-directed or mark directed behaviours, their understanding cannot be quantified as easily.

***Authors’ response:*** *We completely agree with these points. However, as both whales exhibited the progression from contingency testing to exhibiting a variety of self-directed behaviors indicating MSR, and one beluga passed the mark test, we have described, quantified and reported the empirical evidence we have documented and related it to what has been reported in the literature for the other captive aquatic species tested that has demonstrated the capacity for MSR, the bottlenose dolphin.*

It is also important to situate these findings within ongoing methodological debates and differences in opinion in MSR literature. Different MSR researchers often have varying thresholds for evidence of MSR capacity in a species, often limiting evidence to great apes, and select corvids. Lack of replication within an individual, and between individuals is typical for MSR research, and requires detailed methods as seen in this paper so that the full context of the individual animals tested can be understood by readers. In this case, while replication was limited within and between individuals, the response occurred under controlled conditions, with careful and justified behavioural categorisations strengthening its interpretive weight.

***References cited in authors’ responses to reviewers:***

*Gallup, GG Jr. (1970). "Chimpanzees: Self recognition". Science. 167 (3914): 86–87.*

*Morrison R, Reiss D (2018). Precocious development of self-awareness in dolphins. PLoS ONE 13(1): e0189813* [*https://doi.org/10.1371/*](https://doi.org/10.1371/) *journal.pone.0189813*

*Povinelli DJ, Rulf AB, Landau KR, Bierschwale DT. (1993.) Self-recognition in chimpanzees (Pan troglodytes*

*Distribution, ontogeny, and patterns of emergence. J Comp Psychol. 107:347±372. PMID:*

*8112048*

*Reiss, D. & Marino, L. (2001). Mirror self-recognition in the Bottlenose Dolphin: A case of cognitive convergence, Proceedings of the National Academy of Sciences, Vol.98. No. 10. (5937-5942).*
